# Pediatric Dental Management for Children With Special Health Care Needs and Disabilities: Short title: Pediatric Dentistry and Special Health Care Needs

**DOI:** 10.31661/gmj.v15i.4270

**Published:** 2026-07-08

**Authors:** Safa Saeed, Myle Akshay Kiran, Reema Sultan Alshahrani, Rahaf Saud Alobaidi, Lena Abdulrahman Alotai, Fatimah Farhan Almalki, Fahad Khalid Almasoudi, Hadeel Ali Almalky, Lama Alhatem, Hadeel Horais Alzahrani, Amirah Aldowish, Randa Abdulaziz Aleyoni, Setah Abothnin, Rana Abdullah Almaiman, Leen Alzaaqi

**Affiliations:** ^1^ Department of Dentistry, Oral and Maxillofacial Surgery and Diagnostic Sciences, King Saud bin Abdulaziz University for Health Sciences, Saudi Arabia; ^2^ Department of Scientific Clinical Research and Pharmacology, Alternative Medicine, Hospital and Health Care Administration, Acharya Nagarjuna University/National Institute of Medical Science, Pratista, Andhra Pradesh, India

**Keywords:** Pediatric Dentistry, Disabled Children, Special Health Care Needs, Oral Health, Behavior Guidance, Teledentistry

## Abstract

**Background:**

Children with special health care needs and disabilities experience a disproportionate burden of preventable oral disease, unmet dental needs, behavioral barriers to treatment, and fragmented access to specialist dental services.

**Materials and Methods:**

This scoping review mapped contemporary evidence on pediatric dental management strategies for children with special health care needs, with emphasis on prevention, behavior guidance, interdisciplinary care, teledentistry, and service delivery. A structured search was planned across PubMed/MEDLINE, Scopus, Web of Science, Embase, the Cochrane Library, Google Scholar, ClinicalTrials.gov, and grey-literature sources for English-language evidence published from January 2015 to August 2025. Evidence was charted using a population-concept-context framework and synthesized narratively according to source type and clinical theme.

**Results:**

The final evidence map comprised 20 topic-relevant sources, including systematic reviews, observational studies, qualitative studies, educational program reports, narrative reviews, textbooks, and expert consensus documents. No randomized controlled trial was verified among the cited sources; therefore, the manuscript was framed as a scoping review rather than an effectiveness-focused systematic review. Four major themes were identified: preventive and caregiver-mediated oral-health support, individualized behavior guidance and sensory adaptation, interdisciplinary medical-dental collaboration, and technology-enabled access, including teledentistry.

**Conclusion:**

Current evidence supports integrated, family-centered, function-based, and prevention-oriented dental care for children with special health care needs and disabilities. However, the evidence base remains limited by heterogeneous designs, provider-centered outcomes, small samples, narrative sources, and limited comparative child-outcome data. Future research should prioritize prospective multicenter studies using standardized clinical, behavioral, access-related, and quality-of-life outcomes.

## Introduction

Oral health is an essential component of general health, development, nutrition, communication, and quality of life during childhood [1, 2]. Children with special health care needs and disabilities are at increased risk of dental caries, gingival inflammation, traumatic dental injury, delayed dental attendance, and unmet oral-health needs because of medical complexity, functional limitations, behavioral challenges, sensory sensitivities, communication barriers, caregiver burden, and limited access to trained dental providers [3, 4, 5, 6, 7, 8]. The term children with special health care needs refers to children who have, or are at increased risk for, chronic physical, developmental, behavioral, emotional, or medical conditions and who require health and related services beyond those generally required by children [9, 10].

In pediatric dentistry, this population includes children with autism spectrum disorder, cerebral palsy, Down syndrome, epilepsy, intellectual disability, psychiatric conditions, congenital disorders, and chronic systemic diseases [8, 9]. For many of these children, conventional dental pathways are insufficient because dental care must be adapted to medical status, developmental profile, sensory tolerance, communication style, behavioral needs, and family context [9, 10].

Recent literature emphasizes a shift from procedure-centered care toward prevention-oriented, family-centered, function-based, and interdisciplinary models [1, 4, 7]. Medical-dental integration has been proposed as a strategy to reduce missed opportunities for oral-health screening, referral, prevention, and continuity of care among children and youth with special health care needs [10]. Function-based assessment has also been recommended because diagnosis alone may not adequately predict cooperation, oral-hygiene ability, treatment tolerance, or need for sedation or general anesthesia [4, 7]. In children with autism spectrum disorder, sensory adaptation, visual supports, desensitization, caregiver participation, and individualized communication strategies are increasingly emphasized as essential components of dental management [5, 6]. Teledentistry and digital-health approaches may also support screening, triage, caregiver education, follow-up, and access to care, particularly for children with geographic, mobility, behavioral, or medical barriers to in-person visits [6, 11]. Despite this growing literature, the evidence remains methodologically heterogeneous [9, 10]. Many available sources are narrative reviews, consensus documents, qualitative studies, provider surveys, educational reports, or textbooks rather than controlled intervention studies [11]. Consequently, a conventional systematic review of intervention effectiveness would overstate the certainty of the available evidence [11, 12]. A scoping review is more appropriate for mapping the range, nature, and limitations of current evidence and identifying priorities for clinical practice and future research [21].

This scoping review aimed to map and synthesize contemporary evidence on pediatric dental management strategies for children with special health care needs and disabilities, focusing on preventive, behavioral, interdisciplinary, technology-assisted, and workforce-development approaches.

## Materials and Methods

### Reporting Framework

This scoping review was reported in accordance with the PRISMA Extension for Scoping Reviews and JBI guidance for scoping reviews [21, 22]. The PRISMA-ScR framework was used because the purpose of the review was to map the extent, nature, and characteristics of the available evidence rather than to estimate the effectiveness of a specific intervention. The review question, population-concept-context eligibility framework, evidence-charting variables, and narrative synthesis plan were defined before final evidence classification. The protocol was not prospectively registered.

### Review question

The review question was: What management strategies have been described or evaluated in pediatric dentistry for children with special health care needs and disabilities, and what evidence supports their clinical use? Search strategy A structured search was planned for PubMed/MEDLINE, Scopus, Web of Science, Embase, Cochrane Library, Google Scholar, ClinicalTrials.gov, and OpenGrey. The search period covered January 2015 to August 2025. Search terms combined controlled vocabulary and free-text terms related to pediatric dentistry, special health care needs, disability, behavior guidance, prevention, interdisciplinary care, and teledentistry.

The core search string was: “pediatric dentistry” OR “child dentistry” OR “dental care for disabled” OR “special health care needs” AND “interdisciplinary care” OR “comprehensive dental care” OR “preventive dentistry” OR “behavior management” OR “teledentistry” AND “children” OR “pediatric patients” OR “disabled children”.

Reference lists of relevant reviews were screened to identify additional eligible sources.

### Eligibility criteria

The eligibility criteria were structured using the population-concept-context framework. The population included children and adolescents with special health care needs or disabilities; the concept included preventive, behavioral, interdisciplinary, and technology-assisted pediatric dental management; and the context included clinical, hospital, community, educational, and telehealth settings. The full eligibility framework is presented in Table1. Sources were excluded if they focused exclusively on adults, non-dental interventions, animal or laboratory studies, unrelated emergency or disaster management, or articles without a clear connection to pediatric oral health care for children with special health care needs.

### Evidence selection and charting

Records were imported into reference-management software and duplicates were removed. Titles and abstracts were screened for relevance, followed by full-text assessment. Data were extracted using a standardized evidence-charting form. Extracted items included source identification, study design, population or focus, intervention or management concept, outcomes or contribution, and appraisal notes. The evidence-charting domains are summarized in Table2.

### Appraisal approach

Because scoping reviews primarily map evidence rather than exclude studies based on quality, sources were not excluded solely because of methodological limitations. However, each source was classified by evidence type and appraisal suitability. Systematic reviews were considered suitable for AMSTAR 2-style appraisal; observational studies for adapted observational checklists; qualitative studies for qualitative appraisal tools; and narrative reviews, textbooks, commentaries, and consensus statements were classified as contextual evidence rather than effectiveness evidence.

### Data synthesis

A narrative synthesis was performed. Sources were grouped into four themes: preventive and educational interventions; behavior guidance and psychosocial support; interdisciplinary and integrated care; and technology-assisted care. The synthesis distinguished child-outcome evidence from provider perceptions, educational reports, policy statements, and expert opinion.

## Results

**Figure-1:**
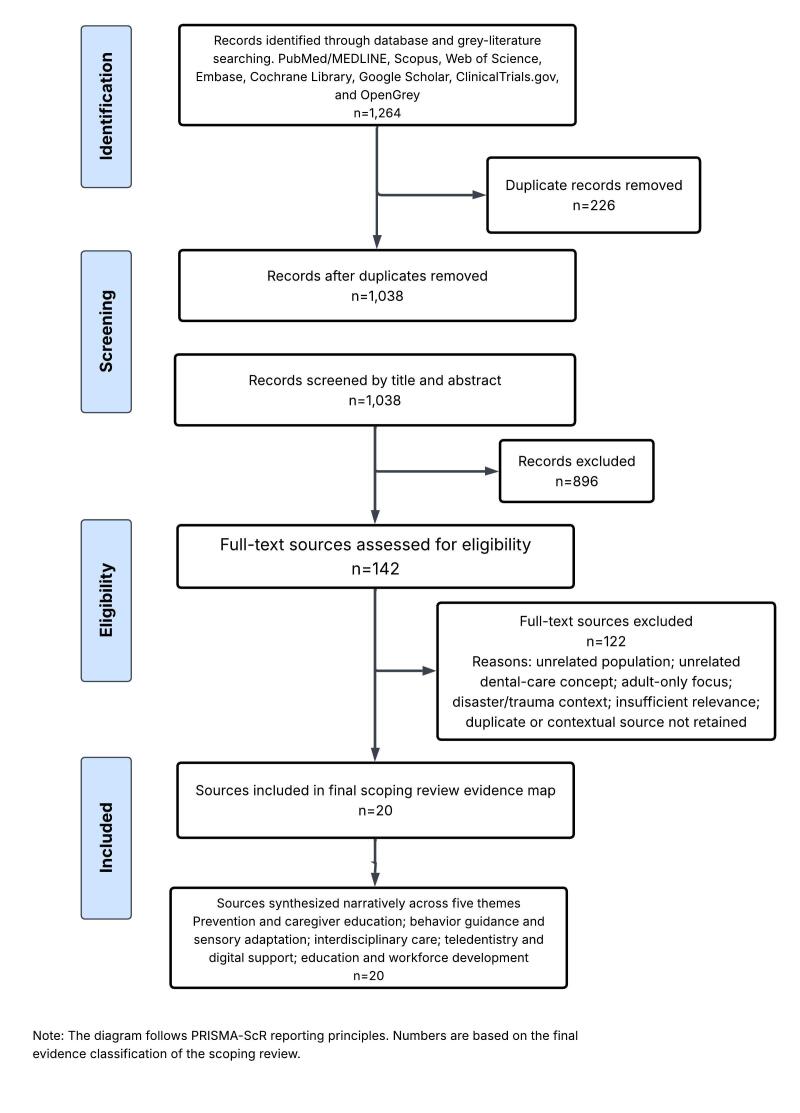
Figure 1. PRISMA-ScR Flow Diagram of Source Identification, Screening, Eligibility Assessment, and Inclusion.

**Table:** Table1. PCC eligibility framework

PCC element	Inclusion criteria	Exclusion criteria
Population	Children and adolescents aged 0-18 years with special health care needs, disability, chronic medical conditions, developmental disorders, or functional limitations	Adults-only populations; animal or laboratory studies
Concept	Pediatric dental management, prevention, behavior guidance, sedation/general anesthesia planning, teledentistry, caregiver education, training, or interdisciplinary care	Non-dental interventions without oral health relevance
Context	Dental clinics, pediatric dental services, hospitals, community care, education programs, telehealth, or integrated medical–dental care	Disaster response, trauma systems, or unrelated healthcare contexts
Evidence type	Empirical studies, systematic reviews, mixed-methods reviews, qualitative studies, educational reports, narrative reviews, textbooks, theses, and consensus documents	Editorials or commentaries with no relevance to pediatric special care dentistry

PCC: population, concept, context

**Table:** Table2. Evidence-charting items

Domain	Extracted data
Source identification	Author, year, country or region, publication type
Population or focus	Child population, disability group, provider group, or service context
Evidence type	Systematic review, observational study, qualitative study, narrative review, textbook, thesis, program report, or consensus document
Management concept	Prevention, behavior guidance, interdisciplinary care, sedation/general anesthesia, teledentistry, education, or policy
Outcomes or contribution	Oral health outcomes, access, cooperation, caregiver role, provider confidence, service model, or evidence gap
Appraisal note	Whether the source supports outcome evidence, provider perspective, contextual background, or expert opinion

**Table:** Table3. Evidence map of included sources

Source	Country/region	Evidence type	Population/focus	Main contribution
Raju et al. [1]	United States	Strategy paper	Children and youth with special health care needs	Describes medical–dental integration as an access strategy
Adeghe et al. [2]	Not country-specific	Perspective/narrative	Children with special health care needs	Proposes screening and access optimization
Ide-Okochi et al. [3]	Japan	Qualitative study	Pediatric dentists	Explores provider perspectives and collaboration needs
Norderyd et al. [4]	Sweden	Observational study	Children with complex disabilities	Links functioning profile with specialist care and anesthesia need
Bikey [5]	Canada	Qualitative thesis	Pediatric dentists treating autistic children	Describes behavior-management decision-making
Zerman et al. [6]	Italy	Narrative review	Children with autism spectrum disorder	Summarizes ASD-focused prevention and management
Norderyd et al. [7]	Sweden	Observational study	Children with disabilities	Applies ICF-CY to oral health and functioning
Ocanto et al. [8]	United States	Program report	Pediatric dentistry residents	Describes ASD-focused resident training
Polli et al. [9]	Brazil	Narrative review	Special-needs dental population	Provides general management overview
Gupta and Hegde [10]	India	Textbook	Children with special needs	Provides clinical background and guidance
Kanani et al. [11]	India	Narrative review	Pediatric dental care	Describes teledentistry applications
Molina et al. [12]	Asia-Pacific	Expert consensus	People with disabilities	Provides regional service and policy recommendations
O’Rourke et al. [13]	Ireland	Narrative review	Dental professionals/students	Discusses special care dentistry education
Kupietzky [14]	International	Textbook	Pediatric dental patients	Provides behavior-management guidance
Molina et al. [15]	International	Systematic review	People with disabilities	Synthesizes caries-management evidence
Dziedzic et al. [16]	Multinational	Commentary	Special care dentistry services	Discusses sedation training and service disruption
López-Velasco et al. [17]	Spain	Systematic review	Children with special needs	Reviews dental care under general anesthesia
Mariño et al. [18]	Chile/international	Workshop report	Dental educators	Discusses pediatric dentistry curriculum reform
Fornefeld et al. [19]	Germany	Cross-sectional survey	Child psychiatrists	Reports perceived oral health needs in psychiatric patients
Erwin et al. [20]	United Kingdom	Mixed-methods systematic review	Autistic children/adolescents	Synthesizes barriers and facilitators to oral healthcare

ASD: autism spectrum disorder; ICF-CY: International Classification of Functioning, Disability and Health for Children and Youth.

**Table:** Table4. Clinical synthesis by theme

Theme	Supporting sources	Practical implication	Evidence limitation
Prevention and caregiver education	[ 1, 2, 7, 9, 10, 15]	Begin preventive care early; tailor fluoride, hygiene, diet, and recall to functional ability and caregiver capacity	Few comparative child-outcome studies
Behavior guidance and sensory adaptation	[ 5, 6, 14, 20]	Use individualized desensitization, visual supports, caregiver preparation, and environmental modification	Heterogeneous behavioral outcomes
Interdisciplinary care	[ 1, 3, 4, 7, 12, 16, 17]	Integrate pediatric dentistry with medicine, anesthesia, psychology, rehabilitation, and caregiver support	Limited implementation and cost-effectiveness evidence
Teledentistry and digital support	[ 6, 11, 20]	Use remote screening, triage, education, and follow-up as adjuncts to in-person care	Needs stronger privacy, equity, and outcome evaluation
Education and workforce development	[ 8, 13, 18]	Include special care dentistry, ASD care, and communication skills in dental training	Mostly provider-confidence or program-description evidence

### PRISMA-ScR Source Selection

The source-selection process is summarized in Figure-1. The initial search identified 1,264 records. After removal of duplicates, 1,038 records remained for title and abstract screening. A total of 142 full-text sources were assessed for eligibility. After evidence verification and editorial reclassification, 20 topic-relevant sources were retained for evidence mapping. One citation concerning orofacial injury management during natural disasters was excluded because it was not cited in the manuscript body and did not match the population, concept, or context of this review.

### Characteristics of included evidence

The included evidence consisted of systematic reviews, observational studies, qualitative studies, educational reports, narrative reviews, textbooks, and expert consensus documents. No randomized controlled trial was verified among the included references. A detailed evidence map of the included sources is provided in Table3.

The most common populations or clinical contexts were children with autism spectrum disorder, children with complex disabilities, children requiring specialist pediatric dental care, and children or youth with special health care needs broadly. Several sources focused on providers rather than children, including pediatric dentists, dental residents, psychiatrists, and dental educators [13, 18, 19]. These sources were retained because they inform service delivery, training, and clinical decision-making, but they should not be interpreted as direct evidence of improved child outcomes. Thematic synthesis The evidence was grouped into five major themes: prevention and caregiver education, behavior guidance and sensory adaptation, interdisciplinary care, teledentistry and digital support, and workforce education. The clinical implications and evidence limitations for each theme are summarized in Table4.

Theme 1: Preventive and caregiver-mediated oral health support Preventive care emerged as the most consistent theme. Sources emphasized early dental referral, caregiver education, individualized oral hygiene instruction, fluoride exposure, dietary counseling, and recall systems tailored to functional ability [1, 2, 7, 9, 10, 15]. Children with special health care needs may depend on caregivers for toothbrushing, diet control, appointment attendance, and adherence to preventive recommendations. Therefore, caregiver-mediated prevention is central to sustainable oral health improvement. The evidence suggests that preventive interventions should be individualized according to diagnosis, motor ability, oral sensitivity, medication use, salivary status, diet, and family capacity. However, many sources provide conceptual or narrative support rather than controlled outcome data. Future studies should measure caries incidence, plaque indices, gingival outcomes, caregiver adherence, oral-health-related quality of life, and treatment avoidance over time.

Theme 2: Behavior guidance and psychosocial support Behavior guidance was a major theme, especially for children with autism spectrum disorder, dental anxiety, developmental disability, communication difficulty, or sensory sensitivity [5, 6, 14, 20]. Commonly described strategies included tell-show-do, modeling, positive reinforcement, desensitization, visual schedules, environmental modification, caregiver presence, audiovisual distraction, and gradual exposure. Provider-centered studies suggest that pediatric dentists adapt behavior-management decisions according to the child’s communication style, previous dental experiences, sensory profile, parental involvement, and urgency of treatment [3, 5]. Sensory-informed modifications, including reduced noise, controlled lighting, predictable sequencing, and visual supports, are particularly relevant for autistic children [6, 20]. These approaches may reduce distress and improve cooperation, but the strength of evidence remains limited by heterogeneous outcomes and few comparative designs. Theme 3: Interdisciplinary and integrated care Interdisciplinary care was repeatedly identified as essential for children with complex disabilities and medically compromised children [1, 3, 4, 7, 12, 16, 17]. Collaboration may involve pediatric dentists, pediatricians, anesthesiologists, speech therapists, occupational therapists, psychologists, nurses, social workers, and caregivers. Function-based approaches may be more useful than diagnosis-based planning because treatment tolerance and general anesthesia need are influenced by communication, mobility, cognition, oral function, and behavioral profile [4, 7]. Hospital-based and specialist care pathways are particularly important for children requiring sedation or general anesthesia [16, 17]. However, sedation and general anesthesia should be embedded within preventive and recall systems rather than used as isolated episodic solutions. Medical–dental integration may improve screening, referral, risk identification, and continuity of care, but stronger implementation studies are needed to determine which models are most effective and scalable [1].

Theme 4: Technology-assisted care and teledentistry Teledentistry was described as a promising adjunct for screening, triage, caregiver education, follow-up, and access support for children who face mobility, geographic, behavioral, or medical barriers to in-person care [11]. Digital platforms may support remote oral hygiene instruction, appointment preparation, monitoring, and caregiver coaching. Video modeling and audiovisual tools may also support behavior guidance before and during dental visits [6, 11, 20]. The current evidence supports teledentistry as a complementary strategy rather than a replacement for clinical examination and treatment. Its usefulness depends on caregiver digital literacy, privacy safeguards, access to devices, quality of intraoral images, reimbursement models, and integration with local referral pathways.

Theme 5: Education and Workforce Development Education and workforce development emerged as an important cross-cutting theme. Pediatric dentists, dental residents, general dental practitioners, and other health professionals require training in special care dentistry, autism-informed care, behavior guidance, communication, sedation referral, caregiver counseling, and interdisciplinary collaboration [8, 13, 18]. Educational reports and curriculum-focused sources suggest that structured exposure to special care dentistry may improve provider confidence, although stronger studies are needed to determine whether training improves patient outcomes [8, 13, 18].

## Discussion

This scoping review maps current evidence on pediatric dental management for children with special health care needs and disabilities. The principal finding is that the literature supports a comprehensive, preventive, and interdisciplinary model of care, but the certainty of evidence is limited by heterogeneity and a shortage of controlled child-outcome studies. The first implication is that prevention should be the foundation of care. Children with disabilities often face repeated exposure to risk factors such as cariogenic medications, feeding challenges, oral motor limitations, caregiver-dependent hygiene, and delayed access to routine dental services. Preventive care should therefore begin early, include caregivers, and be adapted to the child’s functional abilities [1, 2, 7, 10]. The second implication is that behavior guidance should be individualized rather than protocol-driven. Traditional pediatric behavior techniques remain relevant, but children with autism spectrum disorder, intellectual disability, or severe anxiety may require structured desensitization, sensory adaptation, visual communication, and caregiver-supported preparation [5, 6, 14, 20]. These strategies should be documented in the dental record so that future visits remain predictable and consistent. The third implication is that interdisciplinary care is not optional for many children with complex needs. Oral health planning may require medical consultation, medication review, anesthesia risk assessment, nutritional input, speech or occupational therapy support, and behavioral planning [3, 4, 7, 16, 17]. A coordinated model can reduce fragmented care and may improve safety, especially for children who require advanced behavior guidance, sedation, or general anesthesia. The fourth implication is that teledentistry may help close access gaps, particularly for screening, triage, caregiver coaching, and follow-up [11]. However, its role should be evaluated using pragmatic outcomes, including appointment completion, early referral, caregiver satisfaction, treatment avoidance, emergency attendance, and equity of access. This review also identified important limitations in the manuscript’s original evidence classification. Several cited sources were narrative reviews, textbooks, commentaries, or consensus statements rather than empirical studies. Several empirical sources focused on provider perceptions rather than child outcomes. No randomized controlled trial was verified among the cited sources. Therefore, statements claiming seven randomized controlled trials, pooled quantitative effects, or precise reductions in unmet dental need should be removed unless supported by additional verified studies.

### Strengths and limitations

The main strength of this review is its transparent reclassification of the evidence base and its alignment with scoping-review methodology. The review maps not only clinical interventions but also service-delivery, educational, and policy-oriented evidence relevant to pediatric special care dentistry. The main limitation is that the included literature remains heterogeneous and includes several lower-level evidence sources. The review also depends on the accuracy of the available reference set and should be finalized only after full-text verification, database search documentation, and completion of a PRISMA-ScR checklist. Because many included sources are not comparative studies, the review cannot determine the effectiveness of specific interventions or generate pooled estimates.

### Future research

Future research should prioritize prospective multicenter studies of integrated care models for children with special health care needs. Standardized outcomes should include caries incidence, plaque and gingival indices, treatment completion, need for sedation or general anesthesia, behavioral cooperation, caregiver burden, quality of life, cost, and access equity. Implementation studies are also needed to evaluate medical–dental integration, teledentistry pathways, and training programs for pediatric dentists and general dental practitioners.

## Conclusion

Children with special health care needs and disabilities require pediatric dental care that is preventive, individualized, interdisciplinary, and family-centered. Current evidence supports caregiver-mediated prevention, function-based behavior guidance, sensory adaptation, medical–dental collaboration, and selective use of teledentistry. However, the evidence base is dominated by heterogeneous observational, qualitative, narrative, and expert sources rather than controlled trials. Stronger prospective studies are required to determine which integrated management models most effectively improve oral health, access, cooperation, safety, caregiver experience, and quality of life for this vulnerable population.

## Conflict of Interest

The authors declare no conflict of interest.

## AI Disclosure Statement

During the preparation of this manuscript, AI–based language assistance (ChatGPT) was used to support manuscript restructuring, language polishing, grammar correction, and improvement of clarity and flow. The authors reviewed, edited, and approved all AI-assisted content and take full responsibility for the accuracy, integrity, and final version of the manuscript.
